# Companion canines: an under-utilised model to aid in translating anti-metastatics to the clinic

**DOI:** 10.1007/s10585-019-10002-5

**Published:** 2019-11-05

**Authors:** Louise van der Weyden, Mike Starkey, Bushra Abu-Helil, Anthony J. Mutsaers, Geoffrey A. Wood

**Affiliations:** 1 Experimental Cancer Genetics (T113), Wellcome Sanger Institute, Wellcome Genome Campus, Hinxton, Cambridge, CB10 1SA UK; 2 Animal Health Trust, Lanwades Park, Kentford, Newmarket, Suffolk CB8 7UU UK; 3 Department of Clinical Studies, Ontario Veterinary College, University of Guelph, Guelph, ON N1G 2W1 Canada; 4 Pathobiology Department, Ontario Veterinary College, University of Guelph, Guelph, ON N1G 2W1 Canada

The development of anti-metastasis therapeutics has been deprioritised by the pharmaceutical industry, partly due to the lack of short-term measurable clinical outcomes such as tumour shrinkage, and failures in late-stage clinical trials [1]. Companion canines with spontaneous cancers could aid in identifying anti-metastatic targets and clinical drug discovery.

## The dog as a model organism for translational human cancer research

Alongside shared environmental exposures and similar microbiomes [2], the spontaneity and heterogeneity of companion canine cancers arguably has more relevance to human patients than traditional laboratory models, such as rodents. Indeed, the ‘One Health Initiative’ recognises the link between the health of humans, animals, and the environment (http://www.onehealthinitiative.com/). The dog genome is well annotated and the human genome shares more ancestral sequence with the dog than the mouse [3], a reflection of which is that for many cancer-associated proteins there is a greater degree of sequence similarity between the human and canine homologs than between the human and mouse proteins [4]. At the protein level, antibodies used in humans for immunohistochemistry and flow cytometry often also work in dogs [5, 6], whereas mice frequently require an antibody specific for the mouse protein, further emphasizing the similarity between human and dog genes. Importantly, many canine metastatic tumours show striking aetiological, epidemiological, genetic and histological similarity to their human counterparts [7]. In addition, as is the case for human cancers, the presence of specific immune cell types in the tumour microenvironment of dogs has been linked with prognosis [8–10].

## Advantages of involving companion canines in the development of anti-metastatics

An important challenge in the development of anti-metastatic drugs is target identification. In order for a drug to be developed and trialled, there has to be a molecule worth targeting, and the definition of what constitutes a ‘suitable’ metastatic target is not widely agreed. However, comparison of the molecular differences between the metastatic tumour cell and the primary mass from which it evolved may elucidate potential therapeutic targets. In humans, it is often not possible to easily obtain both primary tumour and metastatic tumour from the same individual, and even more difficult to obtain the latter before any treatment has commenced. Tissue samples from the metastatic tumour as well as the primary tumour of the same animal are often available at post-mortem, although whether or not they are collected depends on the institution and the resources they have available to sustain this as a routine practice. Humane euthanasia of dogs with terminal cancer and declining health is common, and when this takes place at a suitably resourced veterinary college with a pathology department many owners consent to necropsy (autopsy), realising that understanding the cause of death of their beloved pet can help with research trying to prevent the death of other pets.

One advantage of using companion canines in clinical trials of anti-metastatics relates to detecting the presence of metastases. The radiographic modalities for imaging metastases in humans (such as MRI, CT, PET-scan) are the same as that used in dogs and are commonly available at veterinary referral hospitals; these have a dual purpose, in being used to monitor metastatic burden as well as measure response to therapy. As an alternative to imaging-mediated treatment response monitoring, it has recently been demonstrated that dogs with osteosarcoma (OSA) that were free of metastases at diagnosis after having received limb amputation and adjuvant chemotherapy, can develop detectable levels of circulating tumour cells (flow cytometric detection of intracellular collagen 1) [11] in their blood before the development of overt metastases or death. The predictive benefits this offers means the relative success of a drug being trialled could be determined without having to wait for the metastatic lesion to become detectable by imaging and/or the dog to suffer clinical signs of tumour burden. In addition, identification of prognostic markers for metastasis development in dogs may also be translatable, facilitating monitoring of high risk human patients to enable early detection of metastasis.

The demonstration that gene expression signatures of metastatic potential could be detected in primary human tumours [12] lead to the identification of metastasis-associated gene expression signatures by comparison of gene expression in human primary tumours that did and did not metastasise [13, 14]. Veterinary researchers have performed similar comparisons of metastasising and non-metastasising primary tumours to identify molecular genetic and epigenetic events associated with canine tumour metastasis, both as targets for anti-metastatic therapeutics and potential prognostic biomarkers [15–19]. Such studies will expedite the identification of potential targets for drugs aimed at preventing human tumour metastasis by strengthening the ‘candidature’, as a target, of genes that are associated with the metastasis of a given tumour in both humans and dogs.

As outlined above, matched primary tumour and terminal metastases from the same dog can sometimes be obtained. This allows for detailed examination of the metastatic tumour cells and the tumour microenvironment including the immune infiltration present, to gain some understanding why a therapy failed in some individuals but greatly prolonged disease-free survival in others. In pursuing the goal of treating metastatic lesions, we recognize that metastases are different from primary tumours, so we need to know the molecular details of metastases that resisted therapy, and this requires sampling these lesions. It is important to consider that dogs develop metastatic disease spontaneously and all the processes in the invasion-metastasis cascade occur naturally, and within the context of a competent immune system. This contrasts with most rodent models where intravenous, intracardiac, or intraosseous injections of already highly malignant and metastasis-competent tumour cells are used to model metastases. It stands to reason that studying spontaneously occurring metastases in dogs increases the likely relevance of preclinical testing of anti-metastatic agents towards treating metastatic progression in human patients. For human and canine metastatic cancers that have a shared metastasis-associated molecular drug target, and which metastasise to similar sites, the rationale for the preclinical testing of an anti-metastatic agent on canine patients is highly compelling.

## Limitations of dogs in aiding cancer drug development

Whilst there are many reasons why dogs represent a good animal model of cancer in humans, there are a number of issues which continue to preclude the involvement of dogs in cancer drug development.

The identification of molecular drug targets that are shared between human and canine tumours will require the analysis of many more canine tumours. The number of canine tumours that have been subjected to genome-wide molecular profiling are but a small fraction of the number of human tumours that have been analysed.

Further along the drug development pathway, there has been some variability in the rigour of canine oncology clinical trials, and the outcome of such trials may not necessarily inform the outcome of a human clinical trial featuring the same tumour and therapeutic. Although much of the infrastructure of canine clinical trials, such as ethical approval, informed consent, data/sample management, and statistical analysis, is comparable to that of human clinical trials, historically there have been no standardized guidelines for conducting a canine clinical trial and thus no specific quality control and/or quality assurance procedures are required to be incorporated into all veterinary clinical studies. For example, trials with non-randomized cohorts are not uncommon, and may lead to skewed results. Non-randomized (e.g. phase II) clinical trials also occur in human medicine, however successful phase II human trials more often lead to randomized phase III trials compared to veterinary medicine, where historically such large trials are less common. As a result, there is an increasing awareness of the need to establish rigorous, standardized veterinary clinical trial designs to ensure valid results are obtained, and to this end workshops have been set up to establish 'Best Practice Recommendations' [20]. One example of this increased standardization and rigour is the Comparative Oncology Trials Consortium, which is part of The National Cancer Institute in the USA [21]. In addition, the American Veterinary Medical Association (AVMA) maintains a clinical trials website (https://ebusiness.avma.org/aahsd/study_search.aspx), which is similar to www.clinicaltrials.gov. It should be noted that both the AVMA and clinicaltrials.gov are essentially repositories that do not necessarily vouch for the rigour of the trials that are listed, and there is no ‘standard’ for inclusion of a trial in these lists. However, it is anticipated that this growing formalization of infrastructure for companion animal clinical trials will greatly increase the reliability of such trials and promote the widespread acceptance of companion canines as valuable models for oncology clinical trials with translatability to humans.

Whilst the study of a spontaneous, clinicopathologically-similar tumour in a dog is intuitively more relevant to a human tumour than the study of an induced 'model tumour' in a mouse, differences still exist between the species that can impact on the translation of clinical trial results from dogs to humans. For example, the acyclic nucleotide analog (GS-9219), which showed marked cytotoxic effects in human lymphoma and leukaemia cell lines in vitro, was evaluated in dogs with naturally occurring, advanced-stage lymphoma and found to display significant efficacy with an acceptable adverse-event profile [22]. However, in subsequent stage I and II clinical trials in humans with haematological malignancies the drug showed marked toxicity and development was stopped. Consequently, we need to accept that, as with any animal model, there will be occasions when differences between humans and dogs mean that drugs cannot always be shared.

The question is, acknowledging these limitations and differences that exist between the two species, do anti-metastatic clinical drug trials in companion canines represent a viable option that should be more vigorously pursued? Perhaps this question is best answered by looking at what we have learned from anti-metastatics clinical trials in companion canines to date.

## Canine osteosarcoma: a highly relevant model for testing anti-metastatics

The majority (> 95%) of canines with appendicular osteosarcoma (OSA) have microscopic pulmonary metastasis at the time of diagnosis and a median overall survival of 4–6 months without adjuvant chemotherapy. Similar to humans, the treatment for OSA in dogs typically involves resection of the primary tumour (generally amputation) with chemotherapy, which increases median survival to 10–12 months. Unlike humans, OSA is a much more common tumour in dogs with an estimated > 10-fold higher incidence than in humans [23]. Fifty percent (14/28) of trials currently listed on the AVMA clinical trials website pertain to OSA, and it is these highly metastatic canine OSAs that have afforded the opportunity to rapidly progress the development of certain targeted therapeutics in human trials. For example, at the end of 2018, Advaxis licensed HER2-targeted agent ADXS31-164 (ADXS-HER2) to OS Therapies for development in human clinical trials for the treatment of patients with recurrent, completely resected OSA https://advaxis.com/clinical-trials-3/her2-associated-cancers/.

Toceranib phosphate (Palladia^™^) is a tyrosine kinase inhibitor (TKI) that was approved by the FDA in 2009 as the first canine-specific cancer drug for treatment of cutaneous mast cell tumours.^1^ [24] In a clinical trial at the Flint Animal Cancer Centre at Colorado State University, dogs with lung metastatic OSA treated with toceranib and high dose losartan (to suppress the activity of inflammatory monocytes^2^) demonstrated a biological response rate of 50% and a measurable response rate of 30%, with acceptable toxicity (AVMA Animal Health Studies Database study: AAHSD000259). With this success, a second multi-institutional clinical trial was initiated in late 2018 using high-dose losartan and toceranib (AAHSD004794), and at the same time, in conjunction with paediatric oncologists at Children’s Hospital Colorado, a clinical trial was being designed for the use of high-dose losartan and the TKI inhibitor, sunitinib, for paediatric bone cancer patients https://www.csuanimalcancercenter.org/blog/new-hope-for-canines-and-kids-with-bone-cancer.

The apoptosis-promoting drug Procaspase-activating compound 1 (PAC-1) is another example of where success after rigorous evaluation in canine cancer patients paved the way for evaluation of the drug in human clinical trials. As a single agent, PAC-1 has shown considerable activity in canines with lymphoma. However, it also may potentiate other therapies. A PAC-1/doxorubicin combination treatment lead to a biologic response in 3/6 dogs with metastatic OSA, and 4/4 dogs with lymphoma [26], whilst a PAC-1/ temozolomide (TMZ) combination achieved biological responses in 3/3 dogs with glioma [27]. Although there are trials at different Institutes within the USA that are currently recruiting canines with lung metastatic OSA to further assess the effectiveness of the PAC-1/doxorubicin combination, Phase I clinical trials using the PAC-1/TMZ combination in humans with high grade gliomas (glioblastoma multiforme or anaplastic astrocytoma after progression following standard first line therapy) have already begun (ClinicalTrials.gov Identifier: NCT03332355). The hope is now that with such a successful track record, it may be possible to address the effectiveness of these combinations in clinical trials of patients with other types of metastatic cancers.

## Current anti-metastatic clinical trials in dogs

Orally administered rapamycin (Sirolimus™), an mTOR inhibitor, is currently being investigated by the Comparative Oncology Trials Consortium, in many different Universities and centres across Canada and the USA, for dogs with amputation-confirmed OSA and no evidence of metastatic disease. The dogs receive standard chemotherapy (carboplatin chemotherapy), followed (or not) by oral rapamycin, with the primary outcome event being the time until development of metastasis (the trial has completed enrolment and the results are due to be released). The National Cancer Institute is also currently running a multi-centre trial (in Canada and the USA), to evaluate a recombinant, attenuated *Listeria monocytogenes* expressing a chimeric human HER2/neu protein as an adjuvant treatment for dogs with OSA, specifically assessing the anti-metastatic effects of the vaccine compared to standard treatment alone (Trial ID: COTC026).

## Mutual benefits

Of course, the suggestion that companion canines should be used in clinical trials of anti-metastatics must not solely be for the benefit of humans—pet dogs are not just alternative mouse models! Dogs with cancer should be involved in the preclinical testing of an anti-metastatic agent where it has been demonstrated that the canine cancer shares the metastasis-associated drug target with the human cancer that the therapy is ultimately intended to treat. There is the possibility that whilst a drug may show promising results in companion canines, it may later be found to fail in human clinical trials, although one would predict that the overall failure rate of this approach in humans would be less than what is currently seen with conventional pre-clinical models. However, most pet owners would probably agree that developing a new treatment that was ultimately found to only benefit dogs would still be a valuable outcome.

## What is likely to promote increased use of companion canines in anti-metastatics drug development?

A key factor to facilitate increased use of companion canines in anti-metastatic drug development is funding. Funding veterinary research for companion animals can be challenging. It is important to appreciate that whilst they are less expensive than human clinical trials, canine oncology clinical trials are significantly more costly than pre-clinical trials in mice [20]. There are also logistical difficulties in collecting ‘fresh’ versus formalin-fixed canine tumour specimens. Both these factors continue to limit the numbers of canine tumours subjected to genome-wide molecular profiling. However, it is the identification and characterisation, in canine tumours, of potential human tumour metastasis drug targets that is the basis for testing anti-metastasis agents on canine cancer patients. Thus, more appreciation of the clinical benefits of comparative oncology research and the use of companion animals in oncology clinical trials would lead to more financial support for such trials by government, philanthropic organisations and pharmaceutical companies. The recognition that there are advantages to using a One Health approach to developing new anti-metastatic therapies may lead to opportunities to fund such studies by cutting across the entrenched barriers of funding human-only or dog-only research studies and clinical trials and allow integrated projects to be undertaken.

A major hurdle for the development of anti-metastatics in either species is the Response Evaluation Criteria In Solid Tumours (RECIST), which is a set of published guidelines, developed in 2000 and updated in 2009 (RECIST 1.1), that define whether tumours in cancer patients shrink, stay the same, or enlarge during treatment [28]. Applying these response guidelines, the success of a drug in clinical trials relies on the demonstration of tumour shrinkage by X-rays, CT scans or MRI scans, with a confirmatory improvement in clinical outcome, and as such does not consider the capability to inhibit the development of metastasis as a measure of success. Only success in shrinking existing primary or metastatic lesions allows a drug to proceed to clinical trials for use in the adjuvant setting, with the aim of preventing/delaying the development of new metastases. Cancer Research UK, Cancer Research Technology and Cancer Therapeutics CRC Australia formed a Metastasis Working Group with representatives from academia, industry, government and regulatory bodies to develop recommendations on how to tackle the challenges associated with treating metastatic disease and reported that “…successful development of effective anti-metastatic therapies will require the regulatory agencies to work together with researchers, drug developers and statisticians to redefine the clinical development paradigm in order to encourage development of this complex but high-potential category of oncology drugs” [1]. Clinical trials in companion canines could help in this respect by incorporating the prevention of the development of metastases as a measurable clinical outcome that is given consideration when calculating the impact of a therapy. Considering that more than half of all dogs with appendicular OSA will have metastases by one year after diagnosis, the timeline for obtaining meaningful outcomes in an anti-metastatic canine trial is much shorter than in humans. It is hoped that pharmaceutical companies and regulatory bodies would then see the benefits that this offers and consider either (i) repurposing drugs classified as ‘failed’ by RECIST measurements in anti-metastatics clinical trials and/or (ii) change the RECIST measurements to include consideration of the inhibition of development of metastases as an outcome of importance.

Another key hurdle that the development of anti-metastatics must overcome is their high failure rate in human clinical trials, making anti-metastasis drug development appear to be a poisoned chalice. One possible reason for this high rate of failure is the fact that pharmaceutical companies do not routinely test drug candidates in metastatic models before moving them into clinical trials. Therefore, utilising a pathway to human clinical trials that moves from ‘preclinical research’ on cell lines and mouse models to ‘veterinary clinical trials’, utilising companion animals as predictors of human efficacy studies, has been proposed [29]. Such an approach opens the door for companion canines with a spontaneously occurring primary tumour and metastases, or no detectable metastases, to be involved in clinical trials of anti-metastatic drugs. The results of such trials may allow better prioritization of which drugs to take to human trials, and could thus potentially result in fewer failures. With more ‘home runs’ on the board, the prospect of increasing successful anti-metastasis drug development will be more appealing.

## Conclusion

The similarities between the aetiology, pathogenesis and clinicopathological characteristics of human and canine cancers afford a rationale for both cross-species research to expedite the identification of targets for anti-metastatic agents, and the pre-clinical testing of anti-metastatics on canine cancer patients. The generosity and enthusiasm of dog owners to enable comparative research, either through sample donation or engagement in veterinary clinical trials, is a great strength of this strategy. The translational success of the companion canine metastatic OSA model has demonstrated the efficacy of veterinary clinical trials in facilitating the development of anti-metastasis drugs for human patients. Given the paucity of success in human clinical trials of anti-metastatics tested in conventional preclinical tumour models, companion canines with metastatic cancers represent a unique preclinical ‘model’ that is significantly under-utilised.

## Footnotes

1 From the label: Palladia is indicated for the treatment of Patnaik grade II or III, recurrent, cutaneous mast cell tumours with or without regional lymph node involvement in dogs (Zoetis).

2 Losartan is a type I angiotensin II receptor (AT1R) antagonist, however, it has been recently demonstrated to exert anti-metastatic activity by inhibiting CCR2 signalling and suppressing monocyte recruitment, thus is being repurposed for use in cancer immunotherapy [25].
